# Mediation Analysis in Bayesian Extended Redundancy Analysis with Mixed Outcome Variables

**DOI:** 10.1017/psy.2024.13

**Published:** 2025-01-03

**Authors:** Ji Yeh Choi, Minjung Kyung, Ju-Hyun Park

**Affiliations:** 1 Department of Psychology, York University, 4700 Keele St., Toronto, ON, Canada; 2 Department of Statistics, Duksung Women’s University, 33 Samyang-ro, 144-gil, Dobong-gu, Seoul, Republic of Korea; 3 Department of Statistics, Dongguk University, 30 Phildong-ro 1-gil, Jung-gu, Seoul, Republic of Korea

**Keywords:** Bayesian statistics, Extended redundancy analysis, mediation analysis, multivariate regression with mixed types of variables

## Abstract

Extended redundancy analysis (ERA) is a statistical approach to component-based multivariate regression modeling that explores interrelationships among multiple sets of while incorporating regression with a data-reduction technique. The extant models that utilize ERA have assumed the outcome variables with the same data type. Also, ERA models focused on estimating direct pathways only without explicitly addressing mediation effects. In this paper, ERA is extended to handle multiple mediators and mixed types of outcome variables by adopting a Bayesian framework, taking into account correlation structure among all of the outcome variables. The proposed method develops an algorithm that derives the joint posterior distribution of parameters using a Markov chain Monte Carlo algorithm. Simulations and an empirical dataset are provided to illustrate the usefulness of the proposed method.

## 1 Introduction

Regression models, encompassing multiple predictors and outcome variables, are pervasive in the social sciences, where research endeavors seek to comprehend the relationships among various variables (e.g., Aiken et al., 1991; Cohen et al., 2003). The inclusion of numerous variables, particularly as predictors, often introduces a level of dependence among them, potentially resulting in the well-known collinearity issue. Several methodologies have been proposed to tackle collinearity, one of which involves integrating a regression model with a data-reduction technique. Extended redundancy analysis (ERA) is one such technique (e.g., DeSarbo et al., 2015; Hwang et al., 2012; Lee et al., 2016, 2018; Lovaglio & Vacca, 2016; Lovaglio & Vittadini, 2014; Takane & Hwang, 2005; Tan et al., 2015).

In ERA, a component is derived from a set of predictors as a weighted composite, maximizing the explained variance in the outcome variables. From a technical standpoint, ERA can be seen as a special case of structural equation models (SEM). This classification arises from ERA’s specification of a formative relation between the predictor set and each component and its exploration of the relationship between the constructed components and outcome variables.

To date, the majority of regression models employing data-reduction techniques, including ERA, have typically assumed that the outcome variables share the same data type, such as being either all continuous or all binary. However, in practical scenarios, encountering multivariate outcome data with diverse types is common—examples include mixed measurements involving both continuous and categorical outcomes. In such instances, we would no longer assume a simplified correlation structure such as compound symmetry or independence, which were conventionally used for most applications with multivariate data in behavioral and social sciences studies (e.g., Henningsson et al., 2001; Lovaglio & Vittadini, 2014).

Moreover, the application of mediation analysis has seen a growing prevalence across diverse fields, including psychology, sociology, economics, and other social sciences (e.g., Bullock et al., 2010; MacKinnon & Fairchild, 2009; Selig & Preacher, 2021; VanderWeele, 2016). According to Pieters (2017), mediation plays a crucial role in theory development, serving as an indispensable tool for assessing potential intermediate effects through intervening variables, referred to as mediators. It is conceptualized as a third-variable effect that illuminates the relationship between a predictor variable and an outcome variable (e.g., Baron & Kenny, 1986; Preacher & Hayes, 2008). Researchers frequently utilize latent variables with SEM framework to investigate and validate theoretical models that encompass both direct and indirect pathways among variables. This involves examining how the influence of a predictor on an outcome variable operates through intermediary mediators. Despite these advancements, extant ERA models have given limited attention to explicating and analyzing such mediation effects.

This paper presents a Bayesian extension to ERA, expanding its application to facilitate not only the estimation of indirect effects involving multiple mediators but also the accommodation of a diverse array of outcome variable types. Implementing ERA with ordinal variables is challenging in the Frequentist approach due to its reliance on the alternating least squares algorithm. In contrast, the Bayesian approach offers greater flexibility in modeling complex structure, such as ordinal variables, by incorporating prior information and probabilistic reasoning. By leveraging a Markov Chain Monte Carlo (MCMC) algorithm, we derive the joint posterior distribution of key parameters, including indirect effects arising from components influencing outcome variables through mediators. To estimate indirect effects as a distinct set of parameters, we build on the fundamental ERA model specification, constructing components as weighted composites of observed predictors, and formulate a unified objective function encompassing both direct and mediation effects.

Furthermore, to enhance the efficiency of the MCMC algorithm while avoiding constraints on the covariance structure of outcome variables with mixed types, we adopt the assumption that a set of continuous latent variables, which underlies ordinal outcome variables, conforms to a multivariate *t* distribution (Park et al., 2021). This strategy enables the incorporation of correlations with continuous outcome variables, which are presumed to adhere to a multivariate *t* distribution, thereby facilitating a more flexible and realistic modeling approach for mixed-type outcome data.

The proposed method is designed to serve several key objectives. Firstly, it aims to preserve the conceptual associations between predictors and their components, thereby handling multicollinearity in cases where a set of predictors exhibits high levels of correlation. Secondly, it seeks to incorporate the advantageous features of the Bayesian framework into ERA, allowing for the joint modeling of mixed outcome data that might otherwise be overlooked in social science research. Lastly, it provides a viable alternative for exploring potential intermediate effects through mediators within the ERA framework.

The remaining part of this paper is organized as follows. Section 2 first explains ERA with mixed types of outcome variables and then the full version of the proposed method that also includes multiple mediators in detail with parameter estimation. Section 3 provides a simulation study. Section 4 provides an application to illustrate the empirical usefulness of the proposed method. The final section summarizes the implications and possible extensions of the proposed method.

## 2 Method

### 2.1 Extended redundancy analysis

Let 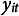 denote the *i*th value of the *t*th outcome variable 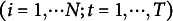 and 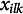 the *i*th value of the *l*th predictor in the *k*th set ( 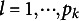 and 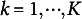 ), where *p_k_* refers to the number of predictors in the *k*th set. Let 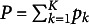 be the total number of predictors in *K* sets. Let 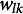 denote a component weight assigned to 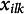 . Let 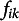 denote the *i*th component score for the *k*th component defined as a linear combination or weighted composite of the predictors in the *k*th set, i.e., 
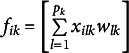
 . Let *a_kq_* denote the *k*th regression coefficient connecting the *k*th component to the outcome variable 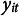 , and 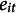 denote the *i*th residual value for 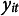 . ERA model can be written as follows:
(1)

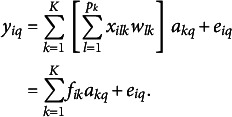



In matrix notation, (1) is re-expressed as

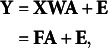

where **Y** is an *N* by *Q* matrix of outcome variables, **X** is an *N* by *P* matrix of predictors, **W** is a *P* by *K* matrix of weights, **A** is a *K* by *Q* matrix of regression coefficients, and **E** is an *N* by *Q* matrix of residuals. For identifiability of **F**, a standardization constraint is imposed on **F** such that 
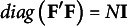
 . More details on the Frequentist ERA can be found in Takane and Hwang (2005) or Choi et al. (2020).

There have been several methodologies described to handle the collinearity problem, among which incorporating regression model with a data-reduction technique is an option. Principal component regression (PCR; Jolliffe, 1982), partial least squares (PLS; Wold, 1975; Wold et al., 1984), and extended redundancy analysis (ERA; Takane & Hwang, 2005) share similarity such that they employ data reduction techniques to transform the predictors into a new set of uncorrelated underlying or latent constructs, more specifically called components. However, PCR extracts components by maximizing the explained variance of the predictors only without considering their associations with an outcome variable. Subsequently, the components are used as the predictors (to fit a regression model by least squares) in a regression model to predict the outcome variable (e.g., Abdi, 2010; Wehrens & Mevik, 2007). In PCR, because components are extracted independently of a regression model, they may not be optimal in explaining the outcome variable the best.

Different from PCR, PLS and ERA do take into account the association between the predictors and an outcome variable when extracting the components. A major distinct feature that differentiates ERA from PLS is on whether or not a single or unified objective function is derived for parameter estimation. While PLS sets up two different objective functions for extracting components and employing a regression model, respectively, ERA estimates the unknown parameters using a single (global) objective function. Also, ERA allows to handle multiple sets of predictors simultaneously whereas PLS involves only one set of predictors. In this paper, we will focus on the most inclusive form of regression with components, i.e., ERA, employing a unified single objective function.

### 2.2 ERA with mixed types of outcome variables

We consider multiple sets of 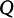 outcome variables which are combinations of a set of 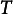 continuous responses and a set of 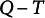 ordinal responses of 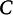 categories. In many cases, outcome variables are correlated, and we need to consider the interdependency among outcome variables in the model regardless of the response structure to avoid biased statistical inference.

#### 2.2.1 Univariate model structure with original regression mean

For continuous outcome variables, we consider a robust regression model to outliers, in the sense that a single out of bounds data point can strongly affect the inference for all the parameters in the model. We are able to reduce the influence of the influential points with considering a longer-tailed family of distributions compared to the normal population model, which allows for the possibility of extreme observations. One of the longer-tailed distributions which are considered frequently is the family of 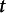 distributions. In our work, we consider the distribution of errors with 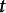 distribution in the place of the normal. Thus, within the multiple regression context, we consider that for the 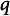 th response:

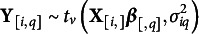
 if 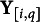 is a continuous response,

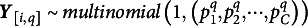
 if 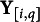 is an ordinal response.

Here the 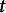 distribution 
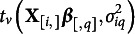
 is characterized by three parameters, center 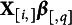 , scale 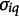 , and a degrees of freedom parameters 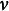 that determines the shape of the distribution, and 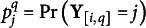 is the probability that the *i*th response for the *q*th outcome variable is the *j*th category with 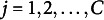 for 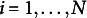 , 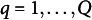 and 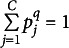 .

For the ordinal response, we consider latent variables with a continuous distributional assumption and cutpoints of the continuous scale for the latent variable. As in Albert and Chib (1993) with the underlying latent observation 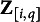 such that

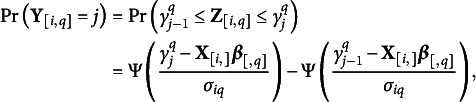

where 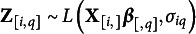 , 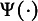 is the cumulative distribution function of the standard logistic distribution, center 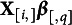 , scale 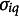 , and cutpoints 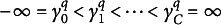 .

Gelman et al. (2014) argued that the 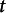 distribution can be considered alternative to logistic and probit regression. Logistic and probit regressions can be nonrobust in the sense that for large absolute values of the linear predictors 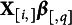 , the inverse logit or probit transformations give probabilities close to 0 or 1. Such models could be made more robust by allowing the occasional misprediction for large values of 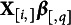 . This form of robustness is defined not in terms of the data 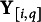 but with respect to the predictors 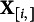 . A robust model can be implemented using the latent-variable formulation of discrete-data regression models which is described above, replacing the logistic or normal distribution of the latent continuous data 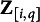 with the model, 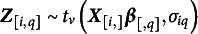 . Gelman et al. (2014) argued that in realistic settings it is impractical to estimate 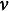 from the data, since the latent data are not directly observed, it is essentially impossible to form inference about the shape of their continuos underlying distribution, so it is set as a low value to ensure robustness. Setting 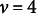 yields a distribution that is close to the logistic, and as 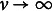 , the model approaches the probit.

In the Bayesian inference, Gibbs sampler computations can often be simplified or convergence accelerated by adding auxiliary variables, and it is called data augmentation. One of the simple but important example of auxiliary variables is the 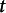 distribution which can be expressed as a mixture of normal distributions. The 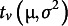 likelihood is equivalent to the model

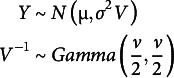

where the 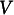 is auxiliary variable that cannot be directly observed.

#### 2.2.2 Multivariate model structure with ERA mean

For the mean structure, we consider ERA with predefined components but unknown weights on predictors for each of components. ERA considers multiple sets (or blocks) of predictors and reduces each set into a component. Such formation is based on some substantive theories or domain knowledge about how certain predictors can be grouped into the same block and aggregated into a component. Each observed variable is hypothesized to be linked to only one component. Accordingly, some elements in 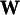 will be constrained to be zero: more specifically, each row of 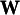 has only one non-zero element (whose component weight will be freely estimated), and the remaining elements in each row will be constrained to zero. Following ERA model specification, the 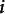 th component 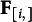 is 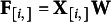 for 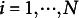 , and hence regression coefficients for the 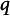 th outcome variable 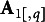 for 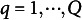 are unidentifiable, so 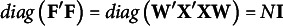 with the 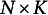 component matrix 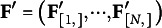 is applied as a standardization contraint for identifiablity (Takane and Hwang, 2005).

For the multivariate logistic distribution, O’Brien and Dunson (2004) proposed a Bayesian models with consideing a multivariate 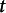 distributed latent variables. The proposed multivariate logistic dstirbution results in the close approximation of the two densities, a multivariate logistic distribution of 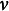 degrees of freedom with center 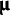 and scale matrix 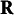 and a multivariate 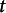 distribution of 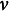 degrees of freedom with center 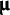 and scale matrix 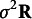 when 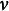 and 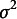 are chosen appropriately. To make the approximation almost exact, O’Brien and Dunson (2004) set 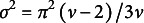 (a value chosen to make the variances of the univariate 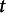 and logistic distributions equail) and set 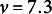 (a value chosen to minimize the integrated squared dsitnace between the univariate 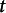 and univariate logistic densities).

Park et al. (2021) introduced a Bayesian methodology for a component-based model that accounts for unstructured residual covariances, while regressing multivariate ordinal outcomes on pre-defined sets of predictors. The proposed Bayesian multivariate ordinal logistic model re-expresses ordinal outcomes of interest with a set of latent continuous variables based on an approximate multivariate *t* distribution based on the method of O’Brien and Dunson (2004). This contributes not only to developing an efficient Gibbs sampler, a Markov Chain Monte Carlo algorithm, but also to facilitating the interpretation of regression coefficients as log-transformed odds ratio.

In this work, we consider multivariate *t* distribution for the residual error on continuous outcomes and multivariate *t* approximation of the latent continuous variables for the ordinal outcomes. We also need to consider the interdependency among outcome variables in the model regardless of the response structure to avoid biased statistical inference. Thus, with an ERA model of 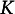 components, for 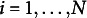 and 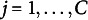

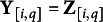
 for a continuous response 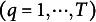

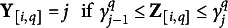
 for an ordianal response 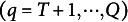 ,

we express our model as following:
(2)

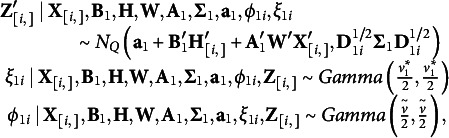

where 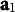 is a length *Q* vector of intercepts, 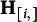 is a 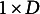 vector of regression predictors, 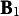 is a 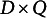 matrix of regression parameters, 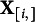 is a 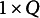 vector of ERA, **W** is a *P* by *K* matrix of weights, 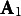 is a 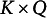 matrix of component coefficients, 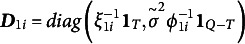 with length *T* vector of 1’s 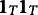 and length 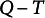 vector of 1’s 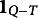 , and 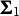 is a 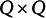 unstructured variance-covariance matrix. Here 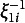 and 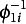 are precision parameters to form multivariate *t* distribution for the *T* continuous responses and 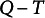 ordinal responses, respectively. As discussed in O’Brien and Dunson (2004), we set 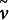 = 7.3 and 
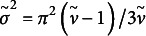
 to make the multivariate *t* distribution approximate the multivariate logistic regression. Because a degrees of freedom parameters 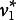 determines the shape of the distribution for continuous outcomes, we treat 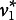 as an unknown parameter which need to be estimated.

### 2.3 The proposed method with mediating effects

A conventional single-level mediation model is expressed as follows:

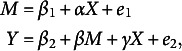

where *M*, *Y*, and *X* denote a mediator, an outcome variable, and a predictor, respectively, with *M* being unobservable while *Y* and *X* are observable. Miočević et al. (2017) included an interaction term ( 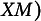 in their model predicting *Y*


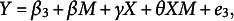

to test if the moderation effect is statistically significant based on Bayesian inference. They considered diffuse (non-informative) priors for the coefficients in their empirical example. In the Markov Chain Monte Carlo Estimation section, they used trace plots to evaluate whether the chains were mixing well, which is indicative of convergence, and provided indexes for diagnosing convergence. Even with the interaction term, there was no issue with the identifiability of model coefficient estimates.

For the indirect or mediation effect of intervening impacts on outcomes, we consider 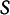 contemporaneous mediators. For 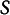 mediators, we consider a multivariate *t* distribution with correlation structure on error term and a component-based ERA structure for the mean trend. As we described above, we express a multivariate *t* distribution with the degrees of freedom 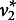 as the hierarchical form of a normal mixture such that
(3)

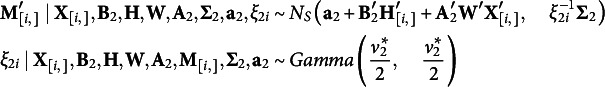

where 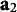 is a length 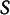 vector of intercepts, 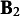 is a 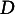 by 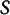 matrix of regression parameters, 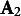 is a 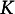 by 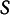 matrix of component coefficients, and 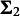 is a 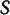 by 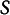 unstructured variance-covariance matrix. We treat the degrees of freedom 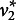 as an unknown parameter for the shape of the distribution.

Similar to the response model in (2), with the *T* continuous responses and 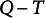 ordinal responses, the multivariate mixed outcomes model with mediators can be expressed as
(4)

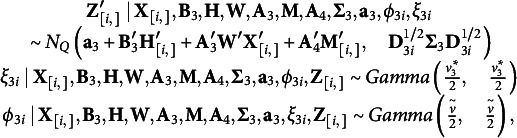

for 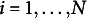 and 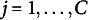 . Here 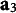 is a length 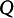 vector of intercepts, 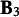 is a 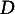 by 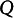 matrix of regression parameters, 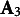 is a 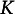 by 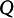 matrix of component coefficients, 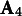 is a 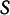 by 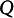 matrix of coefficients relating the mediators to the dependent variables adjusted for the independent variables, 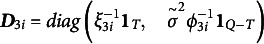 with length 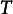 vector of 1’s 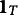 and length 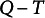 vector of 1’s 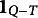 , 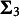 is a 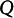 by 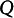 unstructured variance-covariance matrix and 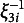 and 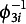 are precision parameters to form multivariate *t* distribution. As we discussed above, 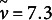 and 
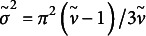
 for the approximation to multivariate logistic regression and we treat 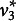 as an unknown parameter for the shape of the distribution for continuous outcomes.

In our model (2)–(4), the pre-determined components 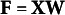 are considered to explain the relationship between predictors and multivariate outcomes (2), to extent to which components changes the mediators (3), and to explain which components are related to the multivariate outcomes adjusted for the mediation (4). Thus the mediated effects can be calculated in two ways as either 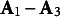 or 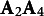 . The value of the indirect effect can be estimated by taking the difference in the coefficients 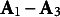 from (2) and (3) corresponds to the reduction in the independent variables effect on the dependence variables when adjusted for the mediator. The product of coefficients method is based on the estimation of (3) and (4) to form the mediated or indirect effect. It can be interpreted as that mediation depends on the extent to which the components change the mediators 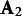 and the extent to which the mediator affects the outcome variables adjusted for the predictors 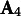 . For the Bayesian inference with rich explanation, we use (3) and (4) models in our work.

### 2.4 Parameter estimation

#### 2.4.1 Prior distributions

In the proposed method, with the likelihood of multivariate *t* distribution formation of the mediating effects in (3) and of the response with mediators in (4), we consider the conjugate distributions for specifying priors on the parameters of interest. For the prior on the weight **W** and the component coefficients **A**, we consider the large valued hyper-parameters of the covariance. For a more flexible Bayesian model, we might be able to consider a prior distribution on the hyper-parameters such as an inverse gamma prios distribution for the conjugacy. However, in our proposed method, to mitigate the effects fo priors, we consider flat priors with large valued hyper-parameters and because of the conjugacy, the convergence of chains could be gauranteed. Note that specifying a prior distribution for the unconstrained covariance matrix 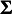 makes our approach different from O’Brien and Dunson (2004), in which unique off-diagonal elements of a correlation matrix are assumed to follow a prior distribution of no specific form. This simple modification leads us more efficient but easier posterior computation, as detailed in Park et al. (2021) for the multivariate ordinal responses. Details about the prior distributions can be found in Appendix A1.

For the prior on 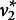 and 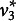 , degrees of freedom parameters of the distribution for mediators and continuous outcomes, we try a uniform density on 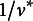 for the range 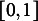 . Gelman et al. (2014) explained that the parameterization in terms of 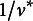 rather than 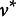 has the advantage of including the normal distribution at 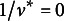 and encompassing the entire range from normal to Cauchy distribution in the finite interval 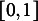 . This prior distribution favors longer-tailed models. We cannot parameterize the 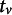 distribution in terms of their variance, because the variance is infinite for 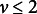 . Instead the interquartile range would be a more reasonable parameter than the curvature for setting up a prior distribution. The interquartile range varies mildly as a function of 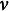 , and we consider the convenient parameterization in terms of mean and variance, and set 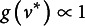 .

#### 2.4.2 Markov chain Monte Carlo

When employing conjugate priors for model parameters, the majority of the full conditional posterior distributions can be derived in closed form. However, closed-form solutions are not available for the posterior distributions of precision parameters and degrees of freedom parameters. Therefore, we update these parameters using a Metropolis step within the MCMC iterations. For the other parameters, Gibbs sampling is a straightforward and easily implementable method. A code programmed in software R (version 4.3.1, R Core Team, 2023) is available on GitHub.

**Figure 1:**
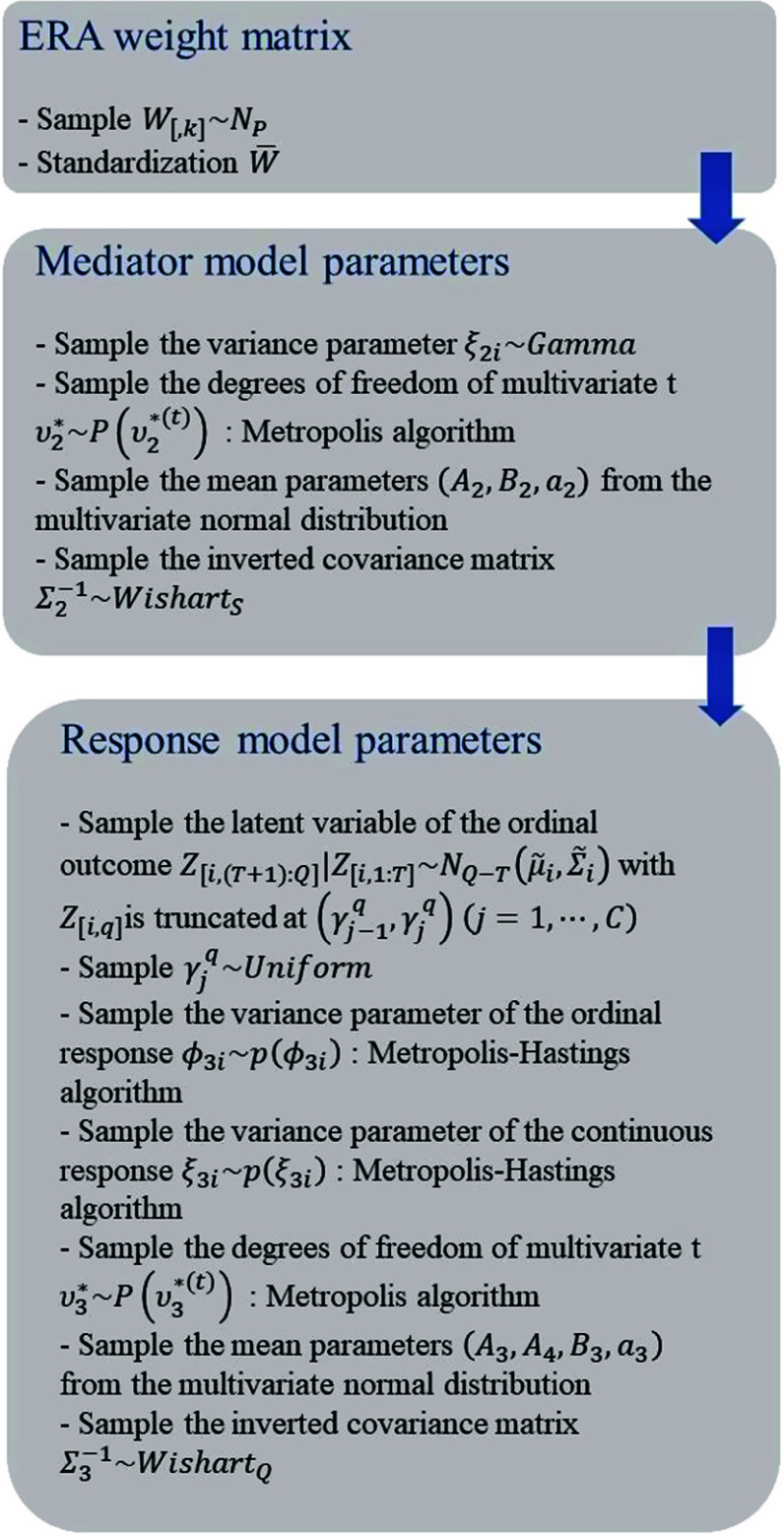
MCMC algorithm to update parameters from the posterior distribution.

To update parameters from the posterior distributions, we choose initial values of parameters from the prior distributions and update parameters based on the full conditional distributions. First, we update the common weight parameter 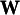 in mediator and response mean terms and standardize it for the identifiability. Next, we update the parameters of the mediator model 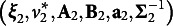 simultaneously and update the latent variable of the ordinal outcomes from the truncated conditional normal distribution. Finally, we update the other parameters in response model 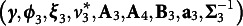 from the conditional posterior distribution of each. We repeat the described steps and the process of the sampling schemes is in Figure 1. Also, the full conditional posterior distributions of parameters and the detailed sampling schemes are in Appendix A2.

There are a few comments to make regarding the proposed algorithm. First, to ensure the identifiability of 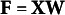 , a standardization constraint is applied to 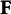 such that 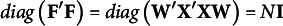 , i.e., a procedure inherent in any ERA model. Second, sampling 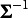 from a Wishart distribution can be easily done in most statistical softwares, for example, with function *rWishart* from the stats library in R. Furthermore, noting that scaled coefficients with 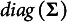 being the diagonal variance matrix corresponding to 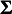 are identifiable and correlations from 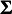 are constrained to be [−1, 1], our proposed algorithm employs the parameter expansion of Gelman et al. (2004), which proves improved convergence of Gibbs sampler in generalized linear models. In contrast, O’Brien and Dunson (2004) draw unique correlations corresponding to 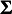 instead using a Metropolis algorithm (Metropolis & Ulam, 1949), for which it is difficult to find a good value of a tuning parameter to control an acceptance probability, especially in a case of sampling multiple variates, often resulting in slow convergence of the algorithm. Third, there is still an additional identifiability problem in intercept 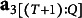 , cutpoints 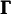 , and 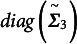 . As Hirk et al. (2019) pointed out, 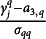 with 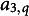 being the *q*th element of 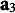 and 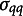 being the *q*th diagonal element of 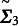 , 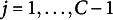 and 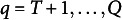 , is only identifiable, and we set for all 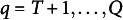 to secure the identifiability of scaled intercepts 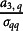 and scaled cutpoints 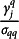 . Such a constraint of 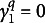 is a common practice in Bayesian logistic regression models for both univariate and multivariate binary outcomes, which is a special case of the proposed multivariate ordinal logistic regression model when 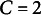 . The fixing of the cutpoint at zero in the process of MCMC is to prevent the identifiability problem of the parameter estimation.

## 3 Simulation study

**Figure 2:**
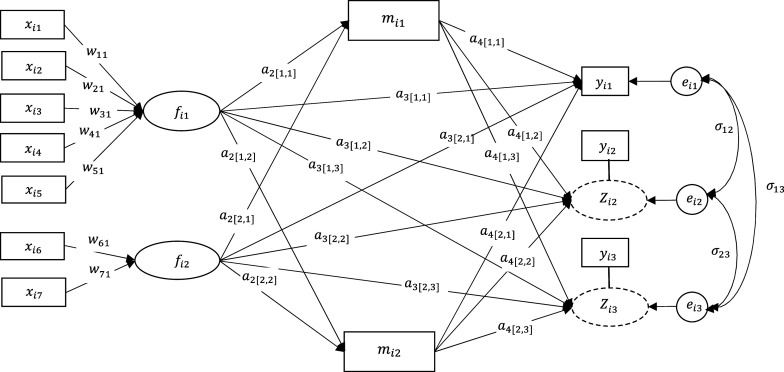
A hypothesized model for the first simulation study (with one continuous and two ordinal outcome variables).

To validate the performance of the proposed method, we conducted simulation studies varying sample sizes of *N* = 100, 300, and 500 for a model with two continuous ( 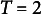 ) and two ordinal outcome variables ( 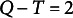 ) using (3) and (4). The hypothesized model had two mediators, and there were no covariate or explanatory variables affecting mediators and outcome variables, as shown in Figure 2. For data generation, we used
(5)

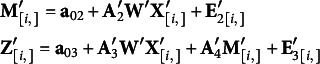

where 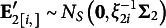 with 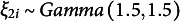 and 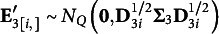 with 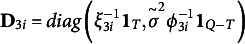 , 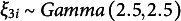 , 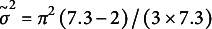 , and 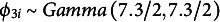 . In (5), the true parameter values were set at 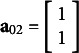 , 
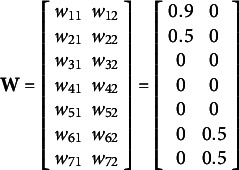
 , 
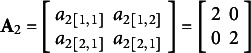
 , 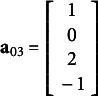 , 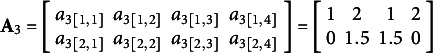 , and 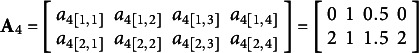 with the covariance matrices 
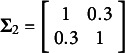
 and 
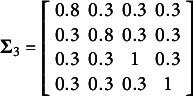
 . In addition, 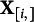 was sampled from a multivariate normal distribution 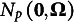 with 
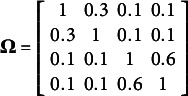
 . Here 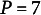 , 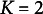 , 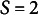 , 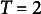 , and 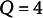 .

Note that the first two element of 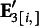 was sampled from a multivariate 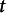 distribution with mean 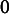 and scale matrix of 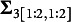 after integrating over 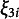 , and the next two elements were sampled from a multivariate 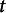 distribution with mean 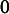 and scale matrix of 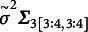 after integrating over 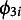 , while these three elements were correlated. A multivariate logistic random variables underlying the two ordinal outcomes were then generated by transforming each of the multivariate 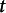 distributed random variables of 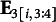 using the following formula proposed by O’Brien and Dunsion (2004), 

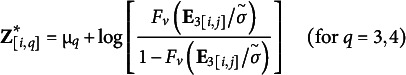

where 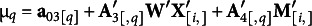 and 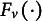 is the cumulative Student’s *t* distribution with 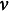 degrees of freedom. The transformation from multivariate *t* variables to multivariate logistic variables is derived in detail in the supplementary information of Kyung et al. (2021). Once 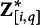 for 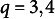 , was generated, two sets of cutoffs were used to obtain the ordinal variables; 
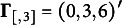
 and 
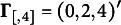
 .

**Figure 3:**
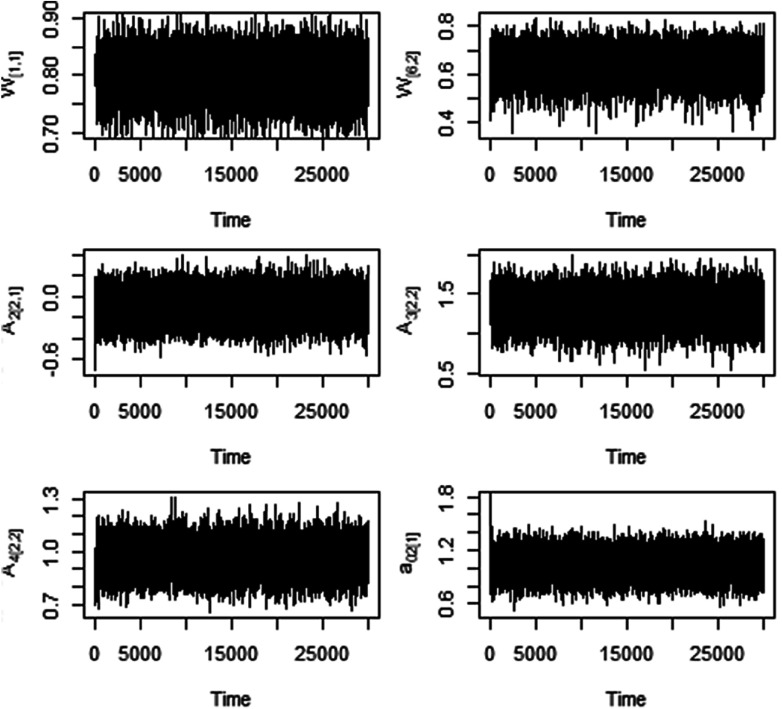
Trace plots of selected parameters for the simulation study with *N* = 100.

For the proposed method, the hyperparameters for diffuse prior distributions were set with all the prior mean parameters such as 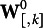 being zero, 
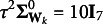
 , 
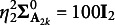
 , 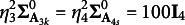 , 
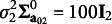
 , 
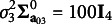
 , 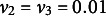 , 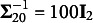 , 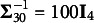 , and the total number of iterations were set at 30,000. Note that these hyperparameters’ setting and the total number of iterations remained the same for all of the analyses conducted in this manuscript (also same for the empirical dataset presented in the next section). Fast convergence and good mixing of the MCMC chain were observed for all parameters of interest across all the simulation scenarios considered. The first 2,000 iterations were discarded as a burn-in period and every fifth posterior sample was used by applying a thinning approach to calculate the posterior means and credible intervals (CI) for the parameters of interest. Relevant simulation code written in R (R Core Team, 2023) is available on GitHub. Fast convergence and good mixing of the MCMC chain were observed for all parameters of interest across all the simulation scenarios considered. As an example, trace plots are presented in Figure 3.

Table 1 presents the results of analyzing the simulated data with two continuous and two ordinal outcome variables varying sample sizes. With increased sample size, posterior mean estimates obtained from the proposed method became on average closer to the true parameter values that were well embedded by narrower corresponding CIs. The residual variances and covariances for the two mediators and four outcome variables ( 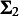 and 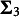 ) exhibited similar patterns: with increased sample sizes, their estimates became closer to the prescribed true values with more precision (resulting in narrower CIs). This indicated that the proposed method is capable of taking into account the dependency among the outcome variables, recovering the true parameter values well.

**Table 1:** Results of the simulation study varying sample sizes (*N* = 100, 300, 500)

			*N* = 100			*N* = 300			*N* =500		
		Parameters		Truth	Post.mean	95CI.low	95CI.up	Post.mean	95CI.low	95CI.up	Post.mean	95CI.low	95CI.up
**W**	*w*_11_	0.9	0.92	0.85	0.99	0.95	0.90	1.00	0.90	0.87	0.94
	*w*_21_	0.5	0.48	0.37	0.59	0.51	0.44	0.58	0.54	0.50	0.59
	*w*_31_	0	−0.01	−0.13	0.09	−0.07	−0.16	0.01	−0.01	−0.07	0.04
	*w*_41_	0	0.00	−0.12	0.11	0.03	−0.05	0.11	0.02	−0.03	0.07
	*w*_51_	0	0.05	−0.07	0.17	0.01	−0.07	0.09	−0.03	−0.09	0.03
	*w*_62_	0.5	0.52	0.43	0.60	0.49	0.43	0.55	0.46	0.42	0.51
	*w*_72_	0.5	0.46	0.37	0.55	0.49	0.43	0.55	0.53	0.49	0.57
**A**_**2**_	*a*_2[1,1]_	2	2.06	1.85	2.27	1.95	1.84	2.07	1.94	1.86	2.03
	*a*_2[2,1]_	0	−0.11	−0.41	0.19	0.03	−0.12	0.19	0.00	−0.12	0.12
	*a*_2[1,2]_	0	0.09	−0.16	0.34	−0.06	−0.17	0.06	−0.08	−0.17	0.00
	*a*_2[2,2]_	2	2.06	1.73	2.38	1.97	1.81	2.13	2.01	1.87	2.13
**A**_**3**_	*a*_*3*[1,1]_	1	0.95	0.53	1.35	0.92	0.72	1.10	0.94	0.78	1.09
	*a*_3[2,1]_	0	0.00	−0.39	0.40	−0.10	−0.32	0.11	−0.06	−0.23	0.11
	*a*_3[1,2]_	2	2.02	1.59	2.43	1.92	1.72	2.11	2.07	1.92	2.22
	*a*_3[2,2]_	1.5	1.63	1.19	2.05	1.42	1.19	1.65	1.43	1.26	1.60
	*a*_3[1,3]_	1	0.84	−0.17	1.95	1.08	0.63	1.54	0.72	0.34	1.09
	*a*_3[2,3]_	1.5	1.63	0.46	2.93	1.04	0.53	1.56	1.28	0.87	1.69
	*a*_3[1,4]_	2	2.93	1.81	4.28	1.59	1.07	2.16	2.03	1.60	2.50
	*a*_3[2,4]_	0	−0.28	−1.40	0.83	0.26	−0.35	0.86	0.14	−0.27	0.55
**A**_**4**_	*a*_4[1,1]_	0	−0.02	−0.20	0.16	0.01	−0.07	0.09	0.00	−0.07	0.07
	*a*_4[2,1]_	2	2.02	1.88	2.16	2.01	1.92	2.10	2.00	1.93	2.06
	*a*_4[1,2]_	1	0.93	0.75	1.12	0.98	0.90	1.06	0.96	0.89	1.03
	*a*_4[2,2]_	1	0.96	0.81	1.11	1.04	0.95	1.13	1.04	0.98	1.11
	*a*_4[1,3]_	0.5	0.83	0.36	1.36	0.37	0.17	0.59	0.50	0.33	0.67
	*a*_4[2,3]_	1.5	1.96	1.20	2.82	1.58	1.27	1.94	1.37	1.14	1.62
	*a*_4[1,4]_	0	−0.56	−0.99	–0.16	0.13	−0.07	0.34	−0.05	−0.22	0.11
	*a*_4[2,4]_	2	2.00	1.31	2.82	1.97	1.59	2.39	1.94	1.65	2.25
**a**_02_	a_02[1]_	1	0.84	0.60	1.08	1.01	0.88	1.14	0.99	0.90	1.09
	a_02[2]_	1	0.92	0.64	1.20	1.16	1.04	1.29	1.01	0.91	1.12
**a**_03_	a_03[1]_	1	1.04	0.80	1.28	0.90	0.73	1.06	0.95	0.82	1.08
	a_03[2]_	0	0.14	−0.11	0.39	0.04	−0.12	0.21	−0.06	−0.17	0.06
	a_03[3]_	2	2.89	1.77	4.18	1.45	0.99	1.94	1.80	1.40	2.19
	a_03[4]_	–1	−0.34	−1.28	0.57	−1.04	−1.61	–0.49	−0.57	−0.99	−0.16
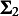	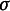 _11_	1	1.14	0.73	1.68	0.97	0.76	1.22	0.98	0.81	1.16
	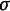 _12_	0.3	0.35	0.05	0.72	0.31	0.17	0.46	0.25	0.14	0.36
	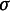 _22_	1	1.52	0.99	2.24	0.95	0.74	1.20	0.99	0.82	1.17
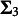	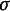 _11_	0.8	0.91	0.58	1.34	1.04	0.83	1.29	0.98	0.84	1.15
	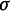 _12_	0.3	0.27	0.04	0.55	0.39	0.25	0.56	0.39	0.29	0.50
	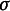 _13_	0.3	−0.01	−0.33	0.30	0.32	0.18	0.48	0.21	0.08	0.33
	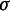 _22_	0.3	0.22	−0.06	0.50	0.09	−0.09	0.26	0.29	0.16	0.41
	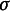 _23_	0.8	1.00	0.61	1.52	1.03	0.83	1.27	0.92	0.78	1.08
	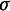 _33_	0.3	0.15	−0.18	0.48	0.33	0.17	0.49	0.10	−0.02	0.22
	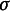 _34_	0.3	0.35	0.01	0.68	0.24	0.06	0.42	0.21	0.08	0.34
	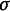 _44_	1	1.00	1.00	1.00	1.00	1.00	1.00	1.00	1.00	1.00

*Note:* Post.mean = posterior mean; 95CI.low = lower bound of the 95% credible interval; and 95CI.up = upper bound of the 95% credible interval. These abbreviated terms remained the same hereinafter.

Table 2 displays the results for indirect effects, whose true values were calculated based on the product term of the prescribed true values for 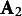 and 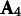 , i.e. 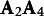 . For instance, the true value of an indirect effect from *F*_2_ on *Y*_1_ via M_1_ was calculated by multiplying the true values of the effect of *F*_2_ on M_1_ and that of M_1_ on *Y*_1_ (i.e., *a*_2[2,1]_ 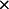 *a*_4[1,1]_). At each iteration of MCMC, the obtained posterior samples on 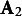 and 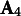 , were used to get the posterior samples for the indirect effects. Based on those posterior samples, we computed posterior means and CIs for the indirect effects. As shown in Table 2, the posterior means of all indirect effect estimates were close to the prescribed true values, and this pattern became salient with narrower CIs as the sample size increased.

**Table 2:** Results of indirect effect estimates obtained from the simulation study varying across sample sizes

			*N* = 100			*N* = 300			*N* =500		
Parameters		Truth	Post.mean	95CI.low	95CI.up	Post.mean	95CI.low	95CI.up	Post.mean	95CI.low	95CI.up
Mediator **M**_**1**_											
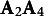	*a*_*2*[1,1]_ 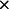 *a*_*4*[1,1]_	0	−0.053	−0.422	0.347	0.010	−0.148	0.177	−0.008	−0.143	0.131
	*a*_*2*[2,1]_ 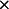 *a*_*4*[1,1]_	0	0.003	−0.033	0.047	0.000	−0.008	0.008	0.000	−0.005	0.005
	*a*_*2*[1,1]_ 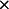 *a*_*4*[1,2]_	2	1.917	1.524	2.359	1.909	1.721	2.110	1.864	1.713	2.024
	*a*_*2*[2,1]_ 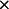 *a*_*4*[1,2]_	0	−0.112	−0.403	0.170	0.031	−0.125	0.188	0.002	−0.115	0.122
	*a*_*2*[1,1]_ 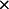 *a*_*4*[1,3]_	1	1.732	0.708	2.869	0.780	0.370	1.209	1.013	0.664	1.377
	*a*_*2*[2,1]_ 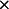 *a*_*4*[1,3]_	0	−0.102	−0.413	0.153	0.013	−0.052	0.083	0.001	−0.064	0.067
	*a*_*2*[1,1]_ 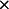 *a*_*4*[1,4]_	0	−1.188	−2.142	–0.326	0.239	−0.165	0.674	−0.110	−0.439	0.243
	*a*_*2*[2,1]_ 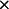 *a*_*4*[1,4]_	0	0.070	−0.103	0.286	0.004	−0.021	0.036	0.000	−0.016	0.014
Mediator **M**_**2**_											
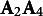	*a*_*2*[1,2]_ 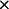 *a*_*4*[2,1]_	0	0.182	−0.316	0.685	−0.118	−0.354	0.124	−0.167	−0.348	0.005
	*a*_*2*[2,2]_ 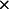 *a*_*4*[2,1]_	4	4.166	3.458	4.894	3.969	3.613	4.336	4.015	3.739	4.302
	*a*_*2*[1,2]_ 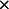 *a*_*4*[2,2]_	0	0.086	−0.150	0.327	−0.061	−0.182	0.063	−0.087	−0.180	0.003
	*a*_*2*[2,2]_ 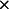 *a*_*4*[2,2]_	2	1.977	1.551	2.437	2.049	1.802	2.301	2.092	1.912	2.281
	*a*_*2*[1,2]_ 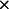 *a*_*4*[2,3]_	0	0.181	−0.318	0.730	−0.098	−0.300	0.103	−0.120	−0.256	0.004
	*a*_*2*[2,2]_ 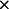 *a*_*4*[2,3]_	3	4.150	2.501	6.062	3.300	2.589	4.048	2.887	2.392	3.437
	*a*_*2*[1,1]_ 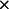 *a*_*4*[1,4]_	0	0.186	−0.324	0.729	−0.119	−0.365	0.129	−0.169	−0.356	0.005
	*a*_*2*[2,1]_ 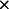 *a*_*4*[1,4]_	4	4.247	2.712	6.047	4.033	3.170	4.943	4.043	3.409	4.730

For comparison, using the same generated datasets from this simulation study, we fitted the same model but excluding the two mediators, that is corresponding to the model in Equation (2) without **H**. Table 3 displays the results without the mediators. Although there were no true values assigned to the direct effects between predictors and outcome variables because the simulation setting was generated with multiple mediators, each of the direct effects could be indirectly inferred from the total effect (i.e., addition of indirect and direct effects) obtained from a model with the mediators. Their direct effects, however, were just rough estimates after controlling for other predictor(s) and mediator(s) (an exact calculation would become only feasible in a setting with single predictor and mediator but without any covariates). When a model was mis-specified by omitting mediators, we found out that the estimates of intercepts **a** and residual variance-covariance matrix 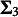 would be more likely to be biased than the weight estimates **W**. The weight estimates remained relatively consistent with the earlier results shown in Table 1. However, unlike the weight estimates, elements of **a** and 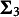 exhibited more bias and susceptibility to the absence of mediators. This indicated that the direct effects alone were insufficient in accounting for all the omitted information associated with the mediators and rather remained as unexplained variation in outcome variables. Consequently, this contributed to biases in the estimates of their intercepts and the residual variance-covariance matrix.

**Table 3:** Results of total effect estimates without the mediators from the simulation study varying across sample sizes

			*N* = 100			*N* = 300			*N* =500		
Parameters		Truth*	Post.mean	95CI.low	95CI.up	Post.mean	95CI.low	95CI.up	Post.mean	95CI.low	95CI.up
**W**	*w*_11_	0.9	0.93	0.87	0.99	0.94	0.91	0.98	0.91	0.88	0.94
	*w*_21_	0.5	0.46	0.37	0.55	0.50	0.44	0.55	0.54	0.50	0.57
	*w*_31_	0	0.00	−0.09	0.09	−0.02	−0.09	0.04	−0.03	−0.08	0.01
	*w*_41_	0	0.08	−0.01	0.17	0.01	−0.05	0.07	0.03	−0.01	0.08
	*w*_51_	0	−0.06	−0.15	0.03	0.03	−0.04	0.09	−0.02	−0.06	0.03
	*w*_62_	0.5	0.54	0.44	0.64	0.50	0.43	0.56	0.46	0.41	0.51
	*w*_72_	0.5	0.44	0.32	0.54	0.49	0.42	0.55	0.53	0.48	0.57
**A**_**2**_	*a*_2[1,1]_	2	–	–	–	–	–	–	–	–	–
	*a*_2[2,1]_	0	–	–	–	–	–	–	–	–	–
	*a*_2[1,2]_	0	–	–	–	–	–	–	–	–	–
	*a*_2[2,2]_	2	–	–	–	–	–	–	–	–	–
**A**_**3**_	*a*_*3*[1,1]_	1	1.24	0.71	1.78	0.79	0.50	1.07	0.81	0.60	1.01
	*a*_3[2,1]_	0	4.08	3.34	4.80	3.73	3.33	4.10	3.83	3.52	4.14
	*a*_3[1,2]_	2	4.08	3.68	4.48	3.76	3.52	3.99	3.86	3.68	4.03
	*a*_3[2,2]_	1.5	3.40	2.83	3.97	3.45	3.13	3.77	3.45	3.20	3.70
	*a*_3[1,3]_	1	1.39	0.99	1.82	0.95	0.74	1.16	1.03	0.86	1.21
	*a*_3[2,3]_	1.5	2.71	1.95	3.52	2.27	1.90	2.65	2.49	2.19	2.81
	*a*_3[1,4]_	2	1.24	0.85	1.69	0.90	0.69	1.12	1.02	0.85	1.20
	*a*_3[2,4]_	0	2.18	1.51	2.90	2.14	1.77	2.52	2.12	1.84	2.41
**A**_**4**_	*a*_4[1,1]_	0	–	–	–	–	–	–	–	–	–
	*a*_4[2,1]_	2	–	–	–	–	–	–	–	–	–
	*a*_4[1,2]_	1	–	–	–	–	–	–	–	–	–
	*a*_4[2,2]_	1	–	–	–	–	–	–	–	–	–
	*a*_4[1,3]_	0.5	–	–	–	–	–	–	–	–	–
	*a*_4[2,3]_	1.5	–	–	–	–	–	–	–	–	–
	*a*_4[1,4]_	0	–	–	–	–	–	–	–	–	–
	*a*_4[2,4]_	2	–	–	–	–	–	–	–	–	–
**a**_02_	a_02[1]_	1	–	–	–	–	–	–	–	–	–
	a_02[2]_	1	–	–	–	–	–	–	–	–	–
**a**_03_	a_03[1]_	1	2.68	2.05	3.29	3.07	2.75	3.38	2.89	2.66	3.13
	a_03[2]_	0	1.65	1.17	2.11	2.12	1.84	2.39	1.90	1.70	2.09
	a_03[3]_	2	2.55	1.91	3.28	1.94	1.63	2.26	2.26	1.99	2.53
	a_03[4]_	−1	0.47	0.00	0.95	0.57	0.30	0.84	0.69	0.49	0.89
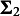	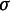 _11_	1	–	–	–	–	–	–	–	–	–
	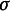 _12_	0.3	–	–	–	–	–	–	–	–	–
	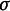 _22_	1	–	–	–	–	–	–	–	–	–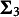	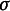 _11_	0.8	8.74	5.98	12.40	7.12	5.73	8.72	6.80	5.79	7.97
	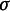 _12_	0.3	5.07	3.28	7.51	4.31	3.32	5.44	4.03	3.33	4.80
	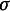 _13_	0.3	2.37	1.73	3.05	2.18	1.86	2.50	1.96	1.73	2.21
	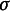 _22_	0.3	2.51	1.93	3.15	2.13	1.80	2.46	2.23	2.01	2.47
	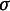 _23_	0.8	4.96	3.37	7.08	5.01	4.00	6.16	4.57	3.90	5.32
	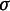 _33_	0.3	1.85	1.41	2.35	1.68	1.40	1.96	1.48	1.28	1.69
	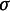 _34_	0.3	1.36	0.86	1.89	1.57	1.29	1.86	1.38	1.18	1.59
	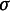 _44_	1	1.00	1.00	1.00	1.00	1.00	1.00	1.00	1.00	1.00

* True values are equivalent to Table 1 as the simulation datasets were generated based on a model with the two mediators.

## 4 An illustrative example

**Figure 4:**
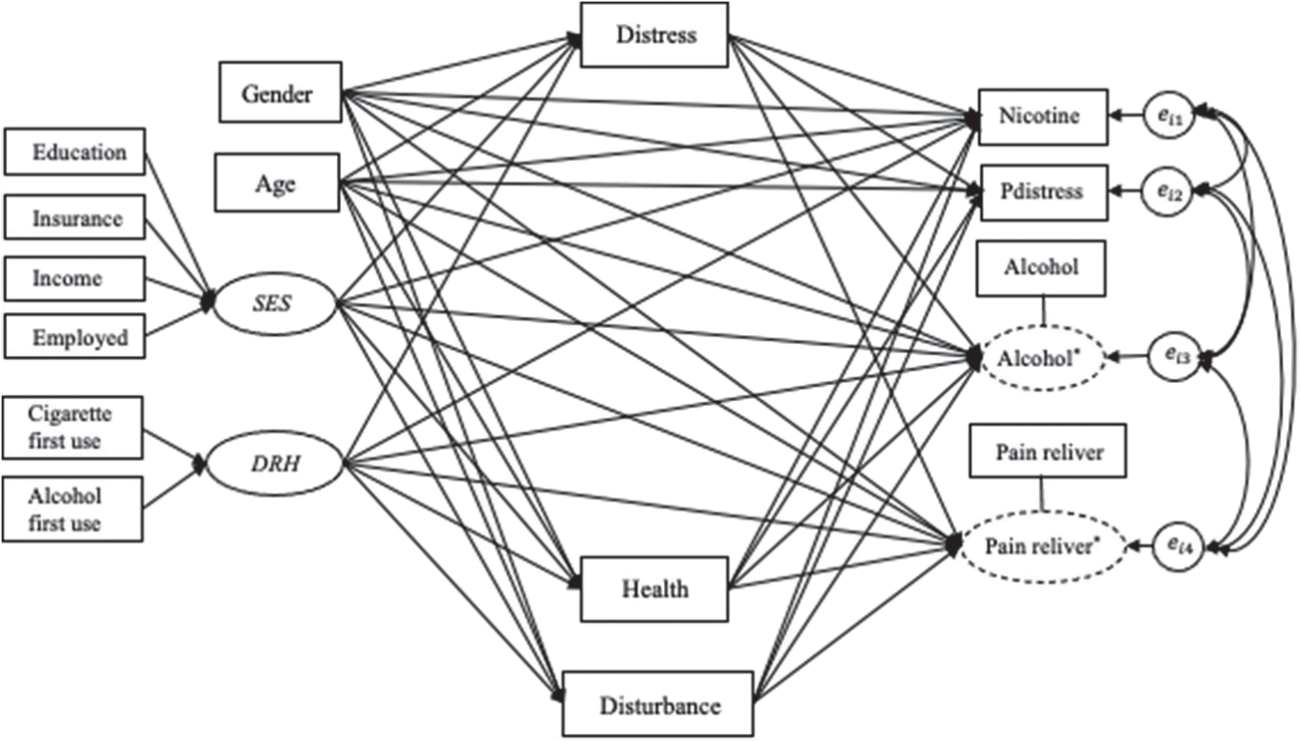
A hypothesized model for the empirical data.

We used a subset of the National Survey on Drug Use and Health (NSDUH) data (Substance Abuse and Mental Health Services Administration (SAMHSA), United States Department of Health and Human Services, 2015), from which 2,347 observations (*N* = 2,347 whose age was 12 or older) responded to a number of questionnaire items concerning substance use and health in 2012. As shown in Figure 4, we constructed two components, i.e., *socioeconomic status* (SES) and *drug history* (DRH). The first component SES was defined as a linear combination of four observed variables: education, insurance, family income, and employment status. The second component DRH was constructed as a linear combination of use of cigarette and alcohol, asking the age of first use. The extent to which participants were dependent on nicotine (measured by nicotine dependence syndrome scale) (nicotine) was used one of the outcome variables. Another continuous outcome variable was measuring past month psychological distress (Pdistress). The remaining two outcome variables were binary variables asking whether or not they have been dependent on alcohol (alcohol) and pain reliever (pain reliver) in the past year. In this example, we examined the effect of SES and drug history on alcohol dependence, psychological distress, and other drug addictions while exploring perceived mental and physical health conditions as potential mediators. Specifically, there were three mediators named distress (measuring the level of psychological distress in the past year excluding the latest past month used for measuring Pdistress), health (measuring the overall perceived health condition), and disturb (measuring the level of psychological impairment and disturbances in social adjustment and behavior). It was hypothesized that the perception of mental and physical health conditions would both mediate the relationship between SES and drug history to alcohol and drug addictions.

**Figure 5:**
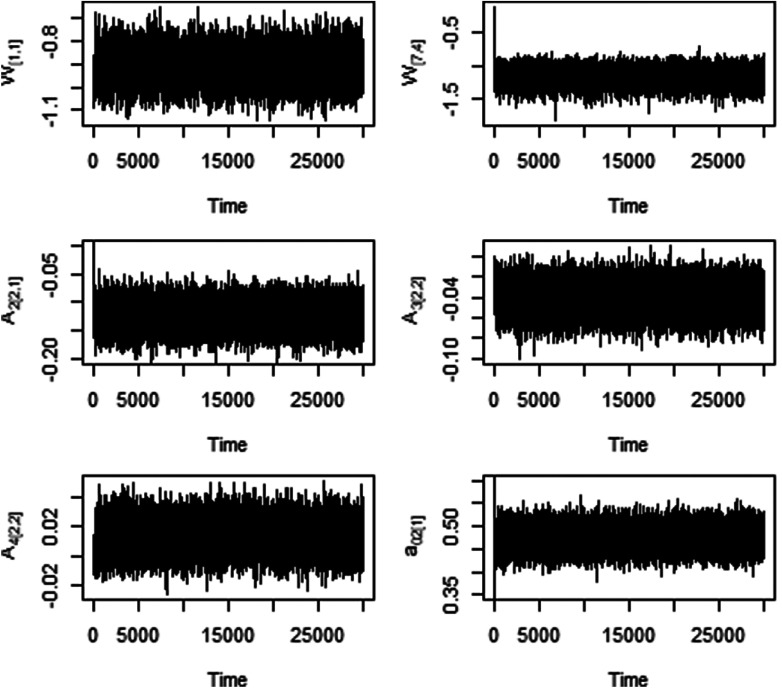
Trace plots of selected parameters for the empirical data.

We fitted this dataset with the proposed method, fast convergence was observed across all parameters of interest as shown in Figure 5. The results for the parameters are presented in Table 4. Looking at the results of direct effects from components on outcome variables given in **A**_**3**_, the two components SES and DRH showed significant and negative direct effects on nicotine addition and pain reliever but not on pain reliver. It suggested that those who have higher socioeconomic status and exposed to cigarette later were less likely to show nicotine dependence (*a*_3[3,1]_ = −0.090, 95% CI = [−0.117, −0.063]; *a*_3[4,1]_ = −0.120, 95% CI = [−0.143, −0.098]) and pain reliver dependence (*a*_3[3,4]_ = 0.383, 95% CI = [0.209, 0.701]; *a*_3[4,4]_ = 0.381, 95% CI = [0.223, 0.646]). The odds of pain reliver dependence was reduced by approximately 62% as both SES and DRH increased by a one-unit. Similarly, the directionality between the two components and past month psychological distress (Pdistress) were all negative. The statistical significance, however, seemed to be present for SES only among the two components. Among the covariates, age showed consistently significant impacts on nicotine, past month distress and pain reliever dependencies (*a*_3[2,1]_ = 0.137, 95% CI = [0.107, 0.167]; *a*_3[2,2]_ = −0.041, 95% CI = [−0.067, −0.015]; *a*_3[2,4]_ = 0.433, 95% CI = [0.205, 0.925]): older people tended to show more dependency on nicotine but less on past month distress and pain reliever. Each additional increase of one year in age was associated with a 56.7% decrease in the odds of being more dependent on pain reliever. Females showed much higher dependency rate on alcohol (1.44 times larger in the odds for alcohol dependency: *a*_3[1,2]_ = 1.439, 95% CI = [1.171, 1.776]) compared to males, but no significant direct impact on nicotine dependence, past month distress and pain reliever dependence.

**Table 4:** Results of fitting the National Survey on Drug Use and Health (NSDUH) data

Parameters		Estimate*		
				Post.mean	95CI.low	95CI.up
**W**	*w*_11_	0.905	0.783	1.025
	*w*_21_	−0.052	−0.143	0.032
	*w*_31_	0.264	0.158	0.378
	*w*_41_	0.384	0.304	0.466
	*w*_52_	1.175	0.958	1.407
	*w*_62_	0.248	0.006	0.469
**A**_**2**_	*a*_2[1,1]_	−0.045	−0.067	−0.024
	*a*_2[2,1]_	−0.124	−0.165	−0.082
	*a*_2[3,1]_	−0.100	−0.133	−0.068
	*a*_2[4,1]_	−0.064	−0.095	−0.034
	*a*_2[1,2]_	−0.078	−0.099	−0.057
	*a*_2[2,2]_	0.034	−0.007	0.075
	*a*_2[3,2]_	−0.113	−0.146	−0.080
	*a*_2[4,2]_	−0.047	−0.077	−0.017
	*a*_2[1,3]_	−0.016	–0.035	0.003
	*a*_2[2,3]_	0.175	0.138	0.212
	*a*_2[3,3]_	−0.204	−0.236	−0.174
	*a*_2[4,3]_	−0.053	−0.080	−0.026
**A**_**3**_	*a*_*3*[1,1]_	0.006	−0.009	0.022
	*a*_3[2,1]_	0.137	0.107	0.167
	*a*_3[3,1]_	−0.090	−0.117	−0.063
	*a*_3[4,1]_	−0.120	−0.143	−0.098
	*a*_*3*[1,2]_	0.006	−0.008	0.019
	*a*_3[2,2]_	−0.041	−0.067	−0.015
	*a*_3[3,2]_	−0.088	−0.109	−0.068
	*a*_3[4,2]_	−0.014	−0.032	0.005
	*a*_*3*[1,3]_	1.439	1.171	1.776
	*a*_3[2,3]_	0.681	0.454	1.030
	*a*_3[3,3]_	1.121	0.814	1.536
	*a*_3[4,3]_	0.806	0.597	1.083
	*a*_*3*[1,3]_	1.408	0.949	2.096
	*a*_3[2,4]_	0.433	0.205	0.925
	*a*_3[3,4]_	0.383	0.209	0.701
	*a*_3[4,4]_	0.381	0.223	0.646
**A**_**4**_	*a*_4[1,1]_	0.085	0.049	0.122
	*a*_4[2,1]_	0.029	−0.006	0.064
	*a*_4[3,1]_	0.089	0.056	0.122
	*a*_4[1,2]_	0.474	0.441	0.506
	*a*_4[2,2]_	0.052	0.021	0.084
	*a*_4[3,2]_	0.116	0.089	0.145
	*a*_4[1,3]_	3.736	2.306	6.061
	*a*_4[2,3]_	2.301	1.484	3.639
	*a*_4[3,3]_	0.876	0.573	1.351
	*a*_4[1,4]_	2.357	0.970	5.973
	*a*_4[2,4]_	4.413	1.892	10.445
	*a*_4[3,4]_	0.362	0.165	0.834
**a**_02_	a_02[1]_	0.758	0.712	0.803
	a_02[2]_	0.555	0.511	0.601
	a_02[3]_	0.551	0.511	0.592
**a**_03_	a_03[1]_	0.475	0.432	0.519
	a_03[2]_	0.143	0.107	0.178
	a_03[3]_	−2.330	−2.912	−1.743
	a_03[4]_	−1.855	−2.845	−0.892
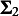	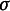 _11_	0.061	0.058	0.065
	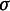 _12_	0.037	0.034	0.041
	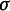 _13_	0.009	0.007	0.012
	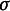 _22_	0.065	0.061	0.069
	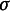 _23_	0.008	0.006	0.011
	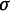 _33_	0.049	0.047	0.052
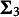	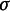 _11_	0.032	0.030	0.034
	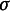 _12_	0.002	0.001	0.003
	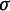 _13_	−0.006	−0.016	0.005
	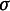 _14_	0.026	0.009	0.043
	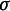 _22_	0.023	0.022	0.025
	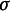 _23_	0.027	0.018	0.036
	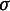 _24_	0.025	0.010	0.038
	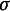 _33_	1.000	1.000	1.000
	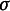 _34_	0.023	−0.117	0.159
	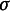 _44_	1.000	1.000	1.000

* The parameters loaded on binary outcome variables were reported by exponentiating the original estimate values. Those reported in odds ratio were displayed with grey shading behind the text. This applied the same to the following table.

The estimates for mediation effects of distress, health, and disturb are presented in Table 5. The indirect effect of distress as a mediator was significant on the two components as well as the two covariates. It was observed that SES and DRH were significant direct predictors of the lower level of nicotine dependence (as shown in A_3_ from Table 3) and also had significant indirect effects through distress (*a*_*2*[3,1]_ 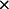 *a*_*4*[1,1]_ = −0.008, 95% CI = [−0.013, −0.004] for the indirect effect of SES on nicotine; *a*_*2*[4,1]_ 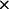 *a*_*4*[1,1]_ = −0.005, 95% CI = [−0.009, −0.002] for the indirect effect of DRH on nicotine). SES and DRH led to lower levels of distress, which in turn yielded a positive impact on nicotine dependence. That is, those who have higher socioeconomic status and were exposed to cigarettes later showed lower level of distress (*a*_2[3,1]_ = −0.100, 95% CI = [−0.133, −0.068]; *a*_2[4,1]_ = −0.064, 95% CI = [−0.095, −0.034]), and this also led to less nicotine addiction (*a*_4[1,1]_ = 0.085, 95% CI = [0.049, 0.122]). This same relationship was also present for the mediation effect of distress on past month distress (*a*_*2*[3,1]_ 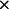 *a*_4[1,2]_ = −0.047, 95% CI = [−0.063, −0.032] for the indirect effect of SES on distress; *a*_*2*[4,1]_ 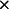 *a*_*4*[1,2]_ = −0.030 95%CI = [−0.045, −0.016] for the indirect effect of DRH on distress). For alcohol dependence, the odds were reduced by about 10% through the mediation effect via distress (*a*_*2*[3,1]_ 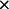 *a*_*4*[1,3]_ = 0.876, 95% CI = [0.817, 0.930] for the indirect effect SES on alcohol; *a*_*2*[4,1]_ 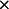 *a*_*4*[1,3]_ = 0.919, 95% CI = [0.870, 0.962] for the indirect effect of DRH on alcohol). While there was a notable and significant indirect effect observed for nicotine, past month distress, and alcohol dependence through a lower level of distress in the past year, the indirect effects on pain reliever were not found to be significant.

**Table 5:** Results of indirect effect estimates obtained from the empirical NSDUH data

Parameters		post.mean	95CI.low	95CI.high
Mediator **Distress**				
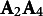	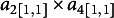	−0.004	−0.007	−0.002
	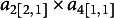	−0.011	−0.017	−0.005
	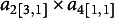	−0.008	−0.013	−0.004
	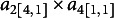	−0.005	−0.009	−0.002
	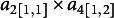	−0.022	−0.032	−0.012
	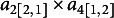	−0.059	−0.078	−0.039
	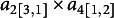	−0.047	−0.063	−0.032
	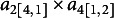	−0.030	−0.045	−0.016
	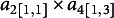	0.942	0.905	0.972
	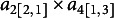	0.850	0.778	0.915
	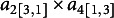	0.876	0.817	0.930
	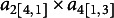	0.919	0.870	0.962
	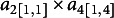	0.962	0.913	1.001
	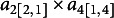	0.900	0.792	1.004
	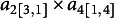	0.918	0.827	1.003
	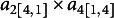	0.947	0.880	1.002
Mediator **Health**				
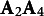	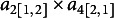	−0.001	−0.003	0.000
	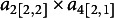	0.016	0.009	0.023
	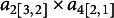	−0.018	−0.026	−0.011
	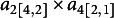	−0.005	−0.008	−0.002
	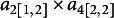	−0.002	−0.004	0.000
	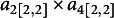	0.020	0.014	0.028
	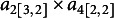	−0.024	−0.031	−0.017
	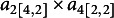	−0.006	−0.010	−0.003
	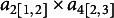	1.002	0.995	1.012
	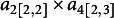	0.977	0.904	1.055
	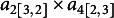	1.027	0.940	1.122
	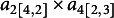	1.007	0.983	1.033
	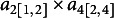	1.016	0.997	1.047
	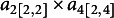	0.837	0.723	0.969
	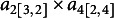	1.231	1.038	1.457
	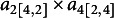	1.056	1.009	1.120
Mediator **Disturbance**				
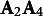	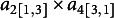	−0.002	−0.005	0.000
	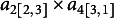	0.001	0.000	0.003
	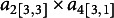	−0.003	−0.008	0.001
	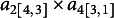	−0.001	−0.004	0.000
	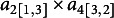	−0.004	−0.007	−0.002
	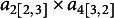	0.002	0.000	0.005
	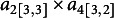	−0.006	−0.010	−0.002
	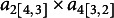	−0.002	−0.005	−0.001
	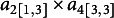	0.937	0.898	0.971
	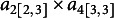	1.029	0.995	1.077
	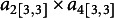	0.910	0.855	0.959
	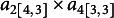	0.962	0.926	0.989
	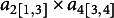	0.891	0.823	0.954
	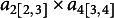	1.052	0.990	1.141
	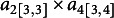	0.846	0.753	0.934
	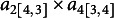	0.933	0.867	0.981

In addition to significant direct effects from SES and DRH on nicotine dependence (*a*_3[3,1]_ = −0.090, 95% CI = [−0.117, −0.063] for SES; *a*_3[4,1]_ = −0.120, 95% CI = [−0.143, −0.098] for DRH), SES and DRH had a significant indirect effect on nicotine dependence (*a*_*2*[3,2]_ 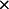 *a*_*4*[2,1]_ = −0.018, 95% CI = [−0.026, −0.011] for the indirect effect of SES on nicotine via health; *a*_*2*[4,2]_ 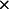 *a*_*4*[2,1]_ = −0.005, 95% CI = [−0.008, −0.002] for the indirect effect of DRH on nicotine via health) and past month distress (*a*_*2*[3,2]_ 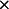 *a*_*4*[2,2]_ = −0.024, 95% CI = [−0.031, −0.017] for the indirect effect of SES on past month distress via health; *a*_*2*[4,2]_ 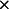 *a*_*4*[2,2]_ = −0.006, 95% CI = [−0.010, −0.003] for the indirect effect of DRH on distress via health) through lower risk for health problems, respectively. The odds of pain reliever dependence increased by 1.231 (*a*_*2*[3,2]_ 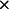 *a*_*4*[2,4]_ = 1.231, 95% CI = [1.038, 1.457]) and 1.056 (*a*_*2*[4,2]_ 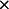 *a*_*4*[2,4]_ = 1.056, 95% CI = [1.009, 1.120]) times, respectively, through the mediation effect via health. Such a pattern was not shown in alcohol dependence. Lastly, one’s perceived level of disturbances in social adjustment and behavior significantly mediated the relationships between the two components and three outcome variables (past month distress, alcohol, and pain reliever dependence). The estimated indirect effects from SES and DRH on distress (*a*_*2*[3,3]_ 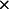 *a*_*4*[3,2]_ = −0.006 (95% CI = [−0.010, −0.002]); *a*_*2*[4,3]_ 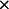 *a*_*4*[3,2]_ = −0.002 (95% CI = [−0.005, −0.001]), alcohol (*a*_*2*[3,3]_ 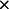 *a*_*4*[3,3]_ = 0.910 (95% CI = [0.855, 0.959]); *a*_*2*[4,3]_ 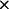 *a*_*4*[3,3]_ = 0.962 (95% CI = [0.926, 0.989]), and pain reliever dependence (*a*_*2*[3,3]_ 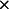 *a*_*4*[3,4]_ = 0.846 (95% CI = [0.753, 0.934]); *a*_*2*[4,3]_ 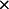 *a*_*4*[3,4]_ = 0.933 (95% CI = [0.867, 0.981]) via disturbances, respectively. SES and DRH showed a negative and significant effect on disturbance, which in turn led to a positive and significant impact on all three outcome variables. Individuals with a lower perceived level of disturbance, influenced by higher SES status and later onset of cigarette consumption, exhibited reduced addictive consumption of alcohol and pain reliever (though not for nicotine dependence). Specifically, when considering odds ratios obtained by exponentiating the corresponding posterior estimates, a one-unit increase in SES and DRH was associated with a 9.00% and 3.80% decrease in the odds of being more dependent on alcohol, respectively, mediated by the level of psychological impairment and disturbance. This statistical significance and interpretation remained consistent for the mediated effect of SES and DRH on pain reliever dependency via disturbance, resulting in a 15.40% and 6.70% decrease in the odds for pain reliever dependency, respectively.

## 5 Concluding remarks

We introduced a multivariate component-based regression model designed to handle mixed types of outcomes and estimate multiple pathways of indirect effects with multiple mediators within a Bayesian framework. The efficacy of our proposed approach was validated through simulated and real data instances. The simulation design intentionally aimed to depict a simplified scenario, characterized by significant discrepancies in actual values, with a particular focus on parameter estimation. In a real-world application, we analyzed a subset of NSDUH data to elucidate how the underlying mechanism of perceived mental and physical health conditions influences the relationship between components (SES and DRH) and drug dependence (nicotine, alcohol, and pain reliever).

In addition to its technical and empirical implications, the proposed method holds the potential for further enhancement in flexibility. An intriguing extension could involve the incorporation of variable selection techniques. While the current conceptualization of the method relies on a predetermined model, there are scenarios where it may be preferable to select an optimal subset of mediators, especially when dealing with a large number of potential mediators. The inclusion of techniques such as lasso (Tibshirani, 1996) or elastic net (Zou & Hastie, 2005) for variable selection could help eliminate irrelevant mediators, thereby improving the interpretability of potential indirect effects within the model.

Moreover, the current proposed method assumes that subjects in the dataset are randomly sampled from the same population. Consequently, it is not possible with the current version to assess differences in effect estimates for different subject clusters using the mediation model with mixed types of outcome data. Considering a recent study proposing a Bayesian approach to ERA with mixture modeling (Kyung et al., 2021), future investigations are warranted to explore the technical and empirical feasibility of incorporating such settings.

## Software availability

To ensure applicability, we have shared an R function along with the simulation code specific to this paper on GitHub (https://github.com/jhppack/BERA_mediation).

## A1 Prior distribution

In the proposed method, we consider the following conjugate distributions for specifying priors on the parameters of interest:

- for
- for
- for
- where
- for
- for
- for
- where
- for  and  .

Here,  refers to a  by 1 column vector containing the weight estimates for the *k*th component,  is a  by 1 column vector containing the component coefficients for the *k*th component affecting the mediators,  is a  by 1 column vector containing the component coefficients for the *k*th component affecting outcome variables when adjusted for the mediator and  is a  by 1 column component coefficients vector of the *s*th mediation variable adjusted for the predictors. Also,  is a  by 1 column vector containing the regression coefficients for the *d*th predictor affecting the mediators and  is a  by 1 column vector containing the regression coefficients for the *d*th predictor affecting the outcomes.  is a  by  matrix of  cutpoints for  ordinal responses.

## A2 Sampling scheme

An overview of the general sampling scheme is as follows.

*ERA structure parameter*

- for  where
   , and  .
- To satisfy the stadardization constraint  , we standardize  such as  .

*Mediator model parameters*

- where  for  .
- (target distribution at  th iteration)
  Metropolis step
  - generate  where  is a candidate distribution function of  .
  - compute acceptance probability  such that
  - set  with probability
- for  where  and
- for  where  and
   with  .
- where  .

- where

*Outcome model parameters*

- The variance matrix of the latent variable of the mixed outcomes will have the form of  where  . The continuous outcomes are observed and we set  , but for the ordinal outcome, we need to update the latent variable while considering the correlation with continuous outcomes. Thus, we consider the conditional distribution of the latent variable of the ordinal outcome given the continuous outcome,  , and it will have a multivariate normal distribution with mean vector
  where  and covariance matrix  . Thus we update the latent variable of the ordinal outcomes from the conditional distribution
  with  is truncated at the left and right by  and  if  for  ,  and  .
- (target distribution at  th iteration) for
  Metropolis–Hastings step
  - generate  where  is a uniform distribution with small number of  and it is a candidate distribution based on previous iteration sample  , thus  is a candidate distribution function of  .
  - compute acceptance probability  such that
  - set  with probability
- (target distribution at  th iteration)
  where  , and it will have a multivariate normal distribution with a mean vector
  with  and covariance matrix  for  .
  Metropolis–Hastings step
  - generate  where  is a uniform distribution with small number of  and it is a candidate distribution based on previous iteration sample  , thus  is a candidate distribution function of  .
  - compute acceptance probability  such that
  - set  with probability
- (target distribution at  th iteration)
  Metropolis step
  - generate  where  is a candidate distribution function of  .
  - compute acceptance probability  such that
     .
  - set  with probability  .
- for  where  and
- for  where  and
   with
- for  where  and
   with
- where  .
- where
